# A crossbred reference population can improve the response to genomic selection for crossbred performance

**DOI:** 10.1186/s12711-015-0155-z

**Published:** 2015-09-29

**Authors:** Hadi Esfandyari, Anders Christian Sørensen, Piter Bijma

**Affiliations:** Department of Molecular Biology and Genetics, Center for Quantitative Genetics and Genomics, Aarhus University, Aarhus, Denmark; Animal Breeding and Genomics Centre, Wageningen University, Wageningen, The Netherlands

## Abstract

**Background:**

Breeding goals in a crossbreeding system should be defined at the commercial crossbred level. However, selection is often performed to improve purebred performance. A genomic selection (GS) model that includes dominance effects can be used to select purebreds for crossbred performance. Optimization of the GS model raises the question of whether marker effects should be estimated from data on the pure lines or crossbreds. Therefore, the first objective of this study was to compare response to selection of crossbreds by simulating a two-way crossbreeding program with either a purebred or a crossbred training population. We assumed a trait of interest that was controlled by loci with additive and dominance effects. Animals were selected on estimated breeding values for crossbred performance. There was no genotype by environment interaction. Linkage phase and strength of linkage disequilibrium between quantitative trait loci (QTL) and single nucleotide polymorphisms (SNPs) can differ between breeds, which causes apparent effects of SNPs to be line-dependent. Thus, our second objective was to compare response to GS based on crossbred phenotypes when the line origin of alleles was taken into account or not in the estimation of breeding values.

**Results:**

Training on crossbred animals yielded a larger response to selection in crossbred offspring compared to training on both pure lines separately or on both pure lines combined into a single reference population. Response to selection in crossbreds was larger if both phenotypes and genotypes were collected on crossbreds than if phenotypes were only recorded on crossbreds and genotypes on their parents. If both parental lines were distantly related, tracing the line origin of alleles improved genomic prediction, whereas if both parental lines were closely related and the reference population was small, it was better to ignore the line origin of alleles.

**Conclusions:**

Response to selection in crossbreeding programs can be increased by training on crossbred genotypes and phenotypes. Moreover, if the reference population is sufficiently large and both pure lines are not very closely related, tracing the line origin of alleles in crossbreds improves genomic prediction.

**Electronic supplementary material:**

The online version of this article (doi:10.1186/s12711-015-0155-z) contains supplementary material, which is available to authorized users.

## Background

Breeding goals in a crossbreeding system should be defined at the commercial crossbred level. However, selection is often optimized to improve animals within pure lines or breeds [1]. Performance of purebred parents can be a poor predictor of the performance of their crossbred descendants in the presence of non-additive gene action or genotype by environment (G × E) interaction. A number of methods have been proposed as alternatives to pure line selection to obtain greater response to selection in crossbreds. These methods can be classified into three groups: reciprocal recurrent selection [2], combined crossbred and purebred selection (CCPS) [3–5] and genomic selection (GS) [6, 7].

Recent studies have shown that GS can be applied to select purebreds for crossbred performance (CP), [6, 8–10]. Compared to alternative methods such as CCPS, GS can lead to substantially greater response to selection [6, 11], lower the rate of inbreeding [6, 12], and does not require systematic collection of pedigree information between crossbreds and purebreds. Moreover, measuring the phenotypes of crossbred animals at each generation of GS may not be necessary, because in theory, predicted SNP effects can be used over a few generations with limited loss in prediction accuracy [13, 14].

For traits with significant non-additive variance, explicitly including dominance in the GS model may increase response to selection of purebreds for CP. Esfandyari et al. [15] investigated the benefits of GS of purebreds for CP, based on purebred information under two conditions, i.e. a low or high correlation of linkage disequilibrium (LD) phase between the two pure lines. They concluded that a dominance model can be used to increase CP, without using crossbred data. Furthermore, they showed that, if the correlation of LD phase between both pure lines is high, accuracy of selection can be increased by combining both pure lines into a single reference population to predict marker effects.

Accepting that GS is an appropriate tool to select animals for CP raises another question i.e. should marker effects be predicted from pure line or crossbred data. On the one hand, if training is carried out on pure lines for traits with significant non-additive variance and therefore potential heterosis, the purebred performance is not a good predictor of crossbred performance. On the other hand, if training is done on crossbreds, it is necessary to record genotype and phenotype data on crossbreds, which can substantially increase the required investment in the breeding program, since crossbred animals are usually not individually identified and individual performances are not recorded. Furthermore, SNP effects in crossbred animals may be specific to the parental line origin, because the extent of LD between SNPs and QTL can differ between the pure lines. Moreover, LD may not be restricted to markers that are tightly linked to the QTL [6]. Nevertheless, training on crossbred data for GS accounts for genetic differences between purebred and crossbred animals and potential genotype by environment effects, and we expect that it can be beneficial to improve crossbred performance.

Previous studies on the implementation of GS in crossbreeding programs focused either on crossbred [8, 9] or purebred [15] data for prediction of marker effects, without explicitly comparing responses to selection obtained with both methods. For example, Zeng et al. [9] compared additive and dominance models for GS of purebred animals for CP by training only on crossbred animals. Therefore, the first objective of our study was to compare response to selection of crossbreds by simulating a two-way crossbreeding program with either a purebred or crossbred training population under a dominance model. In addition, in the dominance model previously proposed by Zeng et al. [9] for the application of GS in crossbreeding programs, alternate heterozygotes (based on breed origin) were assumed to have the same effect. Thus, the second objective of our study was to compare the benefits of GS of purebreds for CP using a crossbred training population when breed origin of alleles was either accounted for or not in the calculation of breeding values. In other words, this study includes models in which alternate heterozygotes can have different effects.

## Methods

### Scenarios

Response to selection in crossbreds was examined in six different scenarios (Table 1). For all scenarios, breed A acted as sire breed and breed B acted as dam breed. Scenarios differed in the structure of the training population. In Scenario 1, both lines A and B had their own purebred training population (separate). In Scenario 2, animals from both breeds A and B were combined into a single purebred training population (combined). In Scenario 3 and 4, crossbred animals had phenotypes but no genotypes, thus the phenotypes of crossbred animals were linked to the genotypes of the purebred animals to predict marker effects, assuming that pedigree information for both purebred and crossbred animals was available. The difference between Scenarios 3 and 4 was that, for Scenario 3, alleles of heterozygous individuals were not traced back to the purebred line of origin, and thus alternate heterozygotes (i.e. genotype Aa or aA) were considered as identical, whereas for Scenario 4, they could be distinguished. For Scenarios 5 and 6, the training population consisted of crossbred animals with both phenotypes and genotypes and, as for Scenarios 3 and 4, alternate heterozygotes were considered as identical in Scenario 5 but could be distinguished in Scenario 6. In the six scenarios presented in Table 1, breeds A and B shared a common ancestor 300 generations back, which means that each breed had 300 generations of independent breeding.

**Table 1:** Simulated scenarios

Scenarios	Training population structure
Scenario 1	PB Separate (A and B)
Scenario 2	PB Combined (A + B)
Scenario 3	Crossbred (P1)
Scenario 4	Crossbred (P2)
Scenario 5	Crossbred (PG1)
Scenario 6	Crossbred (PG2)

“Separate” means that training was done separately for each pure line; “Combined” means that training was done on a combination of purebred lines A and B; “Crossbred (P1)” means that training was done on crossbred animals with phenotypes and genotype probabilities and it was assumed that the alternate heterozygotes were identical in crossbred animals. “Crossbred (P2)” means that training was done on crossbred animals with phenotypes and genotype probabilities and it was assumed that the alternate heterozygotes could be distinguished in crossbred animals. “Crossbred (PG1)” means that training was done on crossbred animals with phenotypes and genotypes and it was assumed that the alternate heterozygotes were identical in crossbred animals. “Crossbred (PG2)” means that training was done on crossbred animals with phenotypes and genotypes and it was assumed that the alternate heterozygotes could be distinguished in crossbred animals

In order to evaluate the impact of relatedness between both pure lines (measured as the number of generations since they separated) and of the size of crossbred training population on response to selection, additional scenarios were simulated for Scenarios 5 and 6 only in which: (1) the number of generations to the most recent common ancestor between breeds A and B varied as follows 1, 50, 100, 200 or 400 generations and (2) the size of the training population varied with 500, 2000 or 8000 randomly selected individuals. All simulated scenarios were replicated 50 times.

### Population structure

**Fig. 1:**
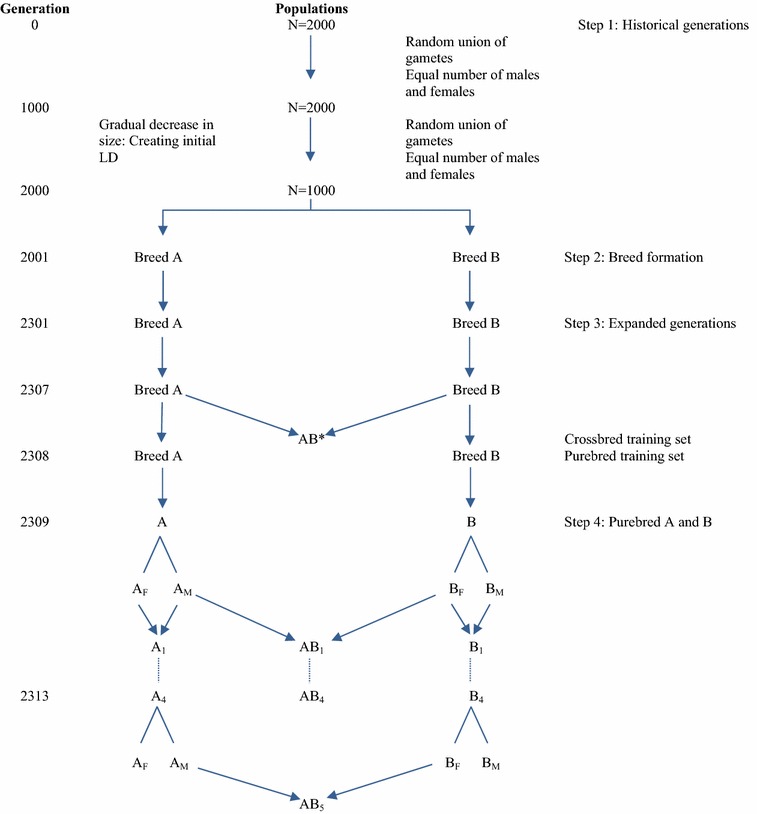
Schematic representation of the simulation steps. The crossbreeding program started in step 4 and consisted of five generations of purebred selection for crossbred performance; the random sample of individuals from the last generation of step 3 (Generation 2308) composed the purebred training set, and crossbred animals (AB*) in generation 2307 composed the crossbred training set; A_M_ and B_M_ represent the selected males of breeds A and B, A_F_ and B_F_ the selected females of breeds A and B; *lines with arrows* denote reproduction, while *lines without arrows* denote selection; the size of the reference population for scenarios with purebred training was 1000 within each pure breed, and 2000 for the scenarios with crossbred training; thus all scenarios had a total reference population size of 2000

The QMSim software [16] was used to simulate a historical population of 2000 generations with a constant size of 2000 individuals for 1000 generations, followed by a gradual decrease in population size from 2000 to 1000 to create initial LD (Fig. 1). The number of individuals of each sex was equal and mating was performed by randomly drawing the parents of an animal from the animals of the previous generation (step 1). To simulate the two purebred recent populations (referred to as breeds A and B, hereafter), two random samples of 100 animals were drawn from the last generation of the historical population and, within each sample, animals were randomly mated for another 300 generations (step 2); 300 generations of random mating for breed formation may seem unrealistic but this was done to simulate two distantly related breeds. In step 3, in order to expand breeds A and B, eight generations were simulated with five offspring per dam. Random mating within each breed was also assumed and no selection was considered in this step.

Since we considered two types of training populations; crossbred and purebred, 400 males and 400 females were selected randomly from generation 7 of step 3 and were randomly mated to produce crossbred offspring, of which 2000 randomly selected animals served as the crossbred training population. Within each pure breed, 1000 randomly selected animals from generation 8 of step 3 were used as the purebred training population for prediction of additive and dominance effects. In subsequent generations (step 4), a two-way crossbreeding program with five generations of selection was simulated. There was no updating of predicted marker effects. The goal was to improve CP through selection in both parental breeds and the selection criterion in both purebred lines was based on genomic estimated breeding values for crossbred performance (GEBVC). The phenotypic mean of crossbreds was computed for each generation of selection (AB_1_ to AB_5_) to evaluate the realized cumulative response to selection.

### Genome and trait phenotypes

A genome consisting of one chromosome of 100 cM with 100 segregating QTL and 1000 SNPs was simulated (Table 2). This small genome size was chosen to limit computing time. In addition, since our objective was to compare CP between simulated scenarios, the absolute level of response to selection and accuracy were not of primary interest. Both QTL and SNPs were randomly distributed over the chromosome. To obtain the required number of segregating loci after 2000 generations, about two to three times as many bi-allelic loci were simulated by sampling initial allele frequencies from a uniform distribution and applying a recurrent mutation rate of 2.5 × 10^−5^. Mutation rates of loci were determined on the basis of the number of polymorphic loci in generation 2000 of the preliminary analysis that were necessary to obtain 1000 polymorphic SNPs and 100 QTL. SNPs and QTL were distinct loci and were randomly drawn from segregating loci, with a minor allele frequency (MAF) higher than 0.05, in generation 2000. It should be noted that this MAF criterion refers to the common ancestral population 300 generations back and thus, lower MAF can occur in the reference population.

**Table 2:** Parameters of the simulated genome

Number of chromosomes	1
Number of markers	1000
Marker distribution	Random
Number of QTL	100
QTL distribution	Random
Initial MAF for markers	0.05
Initial MAF for QTL	0.05
Additive allelic effects for markers	Neutral
Additive allelic effects for QTL	Gamma (0.4,1.66)
Dominance degree for QTL ($h_{i}$)	$N(0.5,0.1)$
Dominance allelic effects for QTL	$d_{i} = h_{i} .\left| {a_{i} } \right|$
Rate of recurrent mutation	2.5 × 10^−5^

The additive effect (*a*) of a QTL, defined as half the difference in genotypic value between alternate homozygotes, was sampled from a gamma distribution (0.4, 1.66). Dominance effects (*d*) were defined as the deviation of the genotypic value of the heterozygote from the mean of the genotypic values of the two homozygotes. Similar to Wellmann and Bennewitz [17, 18], first, dominance degrees at the $i^{\text{th}}$ QTL ($h_{i}$) were sampled from a normal distribution, $N(0.5, 0.1)$, and then dominance effects were calculated as $d_{i} = h_{i} .\left| {a_{i} } \right|$, where $\left| {a_{i} } \right|$ is the absolute value of the additive effect for each QTL. Thus, the absolute magnitudes of additive and dominance effects were not independent, i.e. loci with large additive effects were also more likely to have large dominance effects. Moreover, since the average $h$ was greater than zero, the average dominance effect was greater than zero, indicating directional dominance. The additive and dominance effects were scaled for each replicate of each scenario to reach additive and dominance variances of 0.3 and 0.1, respectively. After scaling, about 12 % of the loci showed overdominance. Furthermore, additive and dominance effects of QTL alleles were assumed to be the same in both breeds. In other words, G × E interactions and epistasis were not simulated. The phenotypes of the trait were simulated by adding a standard normal residual effect to the genotypic value of each animal. The variance of the residual effects was chosen such that broad sense heritability $H^{2}$ of the trait was equal to 0.4. As a result, phenotypic variance ($\sigma_{p}^{2}$), narrow sense heritability $h^{2}$ and dominance variance were equal to 1, 0.3 and 0.1, respectively.

### Prediction of marker effects

The Bayesian ridge regression was used to predict marker effects. We used the BGLR “Bayesian general linear regression” R package developed by Perez and de los Campos [19] and its built-in default rules to set values of hyper-parameters. The following two models were used to predict the genetic effects associated with each marker:

(a) The first model was used for Scenarios 1, 2, 3 and 5, for which alternate heterozygotes (Aa and aA) could not be distinguished. The model used to predict genotypic values was as follows:

$$y_{i} = \mathop \sum \nolimits X_{{AA_{ij} }} g_{1j} + \mathop \sum \nolimits X_{{Aa_{ij} }} g_{2j} + \mathop \sum \nolimits X_{{aa_{ij} }} g_{3j} + e_{i} ,$$

where $y_{i}$ is the phenotypic value of individual $i$ in the training data. For Scenarios 1, 2 and 5, $X_{..ij}$ is an indicator variable of the genotype of individual $i$ at SNP $j$, with $X_{{AA_{ij} }} = 1$ when individual $i$ is AA and $X_{{AA_{ij} }} = 0$ otherwise. Similarly, $X_{{Aa_{ij} }} = 1$ when individual $i$ is Aa and $X_{{Aa_{ij} }} = 0$ otherwise, and with $X_{{aa_{ij} }} = 1$ when individual $i$ is aa and $X_{{aa_{ij} }} = 0$ otherwise. $g_{1j}$, $g_{2j}$ and $g_{3j}$ are the random unknown genotype effects for marker $j$, and $e_{i}$ is the residual effect for animal $i$. The Σ denotes summation over all SNPs $j$.

For Scenario 3, animals in the training population had phenotypes but no genotypes. Therefore, in this scenario, $X_{..ij}$ were genotype probabilities based on the genotypes of parents, rather than indicator variables. To calculate the three genotype probabilities P(AA), P(Aa), and P(aa) for a bi-allelic SNP with two alleles, A and a, for animal $i$, we considered the genotyped sire and dam of the animal. For any genotyped parent, the probability to transmit allele A is P(A) = 1 for the homozygous state (AA), P(A) = 0.5 for the heterozygous state (Aa and aA), and P(A) = 0 for the alternative homozygous state (aa). The probability to transmit allele a is P(a) = 1 − P(A). Thus, based on the genotypes of the parents, the values of *X* were equal to 0, 0.25, 0.5 or 1. For example, if both the sire and dam of animal $i$ were heterozygous (Aa or aA), then the probabilities of observing genotypes AA, Aa and aa in the offspring were equal to 0.25, 0.5 and 0.25, respectively.

(b) The second model was used for Scenarios 4 and 6, for which alternate heterozygotes Aa and aA could be distinguished, and was as follows:

$$y_{i} = \mathop \sum \nolimits X_{{AA_{ij} }} g_{1j} + \mathop \sum \nolimits X_{{Aa_{ij} }} g_{2j} + \mathop \sum \nolimits X_{{aA_{ij} }} g_{3j} + \mathop \sum \nolimits X_{{aa_{ij} }} g_{4j} + e_{i} .$$

For Scenario 6, *X*-elements are the same as for Scenarios 1, 2 and 5. However, since, in this model, alternate heterozygotes Aa and aA could be distinguished in crossbreds, $X_{{Aa_{ij} }} = 1$ when individual $i$ is Aa and $X_{{Aa_{ij} }} = 0$ otherwise, while $X_{{aA_{ij} }} = 1$ when individual $i$ is aA and $X_{{aA_{ij} }} = 0$ otherwise.

For Scenario 4, animals used for training had phenotypes but no genotypes and thus,$X_{ij}$ were genotype probabilities based on the genotypes of parents, rather than indicator variables. Since in this model, alternate heterozygotes Aa and aA could be distinguished in crossbreds, four genotype probabilities P(AA), P(Aa), P(aA), and P(aa) were considered. For example, if the sire and dam of animal $i$ were both heterozygous (Aa) at marker $j$, the probabilities of observing any of the genotypes AA, Aa, aA and aa in a crossbred offspring were all equal to 0.25.

### True and genomic estimated breeding values

The true breeding value for crossbred performance (TBVC) for each animal was calculated as the expected genotypic value in the offspring of a parent carrying a certain QTL-genotype, when this parent was randomly mated to an individual of the other pure line. For crossbred offspring, the expected genotype frequencies of the offspring of a parent depend on the allele frequencies in the other pure line (denoted *ŕ* here). Thus, for animal $i$ from breed $r$, the true breeding value for CP was calculated as:

1 $$TBVC_{ir} = \mathop \sum \limits_{j = 1}^{100} [(X_{{AA_{ij} }} )(p_{{jr^{\prime}}} a_{j} + q_{{jr^{\prime}}} d_{j} )] + [(X_{{Aa\& aA_{ij} }} )(0.5p_{{jr^{\prime}}} a_{j} + 0.5q_{{jr^{\prime}}} d_{j} + 0.5p_{{jr^{\prime}}} d_{j} - 0.5q_{{jr^{\prime}}} a_{j} )] + [(X_{{aa_{ij} }} )( - q_{{jr^{\prime}}} a_{j} + p_{{jr^{\prime}}} d_{j} )],$$

where $X_{{AA_{ij} }} , X_{{Aa\& aA_{ij} }}\, {\text{and}}\, X_{{aa_{ij} }}$ are indicator variables of the genotype at the $j{\text{th}}$ QTL of the $i{\text{th}}$ purebred individual. Thus, $X_{{AA_{ij} }} = 1$ when the genotype is AA and ${\text{zero}}$ otherwise, $X_{{Aa\& aA_{ij} }} = 1$ when the genotype is Aa or aA and 0 otherwise and $X_{{aa_{ij} }} = 1$ when the genotype is aa and 0 otherwise. Moreover, $p_{{jr^{\prime}}}$ and $q_{{jr^{\prime}}}$ are the allelic frequencies (A and a) for the $j^{th}$ QTL in breed $r^{\prime}$. and $a_{j}$ and $d_{j}$ are true additive and dominance effects at the $j{\text{th}}$ QTL. For example, for a parent with genotype AA at locus $j$, a fraction $p_{{jr^{\prime}}}$ of its offspring will have genotype AA, while a fraction $q_{{jr^{\prime}}}$ of its offspring will have genotype Aa. Hence, for locus $j$, the breeding value of this parent equals $(p_{{jr^{\prime}}} a_{j} + q_{{jr^{\prime}}} d_{j} )$, which is e first term of Eq. 1. Equations 1 and 2 are simply the expected crossbred progeny averages for an animal with a certain genotype. These could also have been calculated from Fisher’s average effect [20] for CP, which would yield identical results.

Genomic estimated breeding values were calculated in the same way except that SNP genotypes rather than QTL genotypes, and predicted genotypic effects were used. Thus, for Scenarios 1, 2, 3 and 5, genomic predicted breeding values for crossbred performance (GEBVC) for animal $i$ from breed $r$ was calculated as:

2 $$GEBVC_{ir} = \mathop \sum \limits_{j = 1}^{1000} [(X_{{AA_{ij} }} )(p_{{jr^{\prime}}} \hat{g}_{1j} + q_{{jr^{\prime}}} \hat{g}_{2j} )] + [(X_{{Aa\& aA_{ij} }} )(0.5p_{{jr^{\prime}}} \hat{g}_{1j} + 0.5q_{{jr^{\prime}}} \hat{g}_{2j} + 0.5p_{{jr^{\prime}}} \hat{g}_{2j} + 0.5q_{{jr^{\prime}}} \hat{g}_{3j} )] + [(X_{{aa_{ij} }} )(q_{{jr^{\prime}}} \hat{g}_{3j} + p_{{jr^{\prime}}} \hat{g}_{2j} )],$$

where, $\hat{g}_{1j}$, $\hat{g}_{2j}$ and $\hat{g}_{3j}$ are predicted genotypic effects for SNP genotypes AA, Aa and aA, and aa, respectively.

In Scenarios 4 and 6, for which alternate heterozygotes Aa and aA could be distinguished, GEBVC for animal $i$ from breed $r$ was calculated as:

3 $$GEBVC_{ir} = \mathop \sum \limits_{j = 1}^{1000} [(X_{{AA_{ij} }} )(p_{{jr^{\prime}}} \hat{g}_{1j} + q_{{jr^{\prime}}} \hat{g}_{2j} )] + [(X_{{Aa\& aA_{ij} }} )(0.5p_{{jr^{\prime}}} \hat{g}_{1j} + 0.5q_{{jr^{\prime}}} \hat{g}_{2j} + 0.5p_{{jr^{\prime}}} \hat{g}_{3j} + 0.5q_{{jr^{\prime}}} \hat{g}_{4j} )] + [(X_{{aa_{ij} }} )(q_{{jr^{\prime}}} \hat{g}_{4j} + p_{{jr^{\prime}}} \hat{g}_{3j} )],$$

where $\hat{g}_{1j} , \,\hat{g}_{2j} ,\,\hat{g}_{3j}\, {\text{and }}\hat{g}_{4j}$ are predicted genotypic values of AA, Aa, aA and aa genotypes at the $j{\text{th}}$ marker, respectively.

### Correlation of LD phase between breeds A and B

Correlation of LD phase between breeds A and B was estimated to evaluate the degree of relatedness between the two simulated breeds. To estimate persistence of LD phase between two lines, only the segregating SNPs with a MAF greater than 0 in both breeds were included in the analysis. Persistence of LD phase was estimated following Badke et al. [21] as follows:

$$R_{AB} = \frac{{\mathop \sum \nolimits (r_{ij(A)} - \bar{r}_{A} )(r_{ij(B)} - \bar{r}_{B} )}}{sd(A)sd(B)},$$

where ${\text{R}}_{{{\text{A}},{\text{B}}}}$ is the correlation of phase between ${\text{r}}_{{{\text{ij}}({\text{A}})}}$ in breed A and ${\text{r}}_{{{\text{ij}}({\text{B}})}}$ in breed B, ${\text{r}}_{\text{ij}}$ is the correlation coefficient as a measure of LD for each pair of SNPs, sd(A) and sd(B) are the standard deviations of ${\text{r}}_{{{\text{ij}}({\text{A}})}}$ and ${\text{r}}_{{{\text{ij}}({\text{B}})}}$, respectively, and $\bar{r}_{\text{A}}$ and $\bar{r}_{\text{B}}$ are the average $r_{ij}$ across all SNPs *i* and *j* within interval *p* for breeds A and B, respectively.

## Results

### Purebred-crossbred genetic correlation

The genetic correlation between TBVP and TBVC, which is known as the purebred-crossbred genetic correlation ($r_{pc}$, Wei and van der Werf [22]) was on average equal to 0.78 ± 0.02. Since G × E interaction was not included in the simulations, the deviation of $r_{pc}$ from 1 was purely due to dominance effects and differences in allele frequencies between the two purebred lines.

### Response to selection in crossbreds

**Fig. 2:**
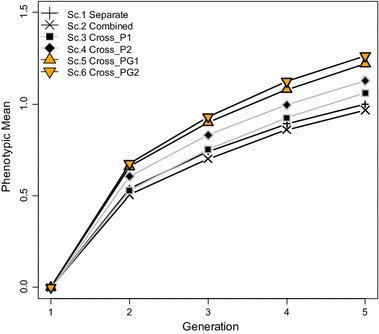
Phenotypic mean of crossbred animals. *Scenario 1* separate training in both breeds A and B. *Scenario 2* training on a combined set of animals from both breeds A and B. *Scenario 3* training on crossbred animals with phenotypes and genotype probabilities and it was assumed that the alternate heterozygotes Aa and aA were identical in crossbred animals. *Scenario 4* training on crossbred animals with phenotypes and genotype probabilities and it was assumed that the alternate heterozygotes could be distinguished in crossbred animals. *Scenario 5* training on crossbred animals with phenotypes and genotypes and it was assumed that the alternate heterozygotes were identical in crossbred animals. *Scenario 6* training on crossbred animals with phenotypes and genotypes and it was assumed that the alternate heterozygotes could be distinguished in crossbred animals; standard errors of phenotypic means ranged from 0.02 to 0.03

The increase in phenotypic mean of crossbred animals was measured over four generations of selection in the six simulated scenarios for which breeds A and B had diverged 300 generations back (Fig. 2). Ranking of scenarios in terms of phenotypic mean of crossbreds showed that training on crossbreds (Scenarios 3, 4, 5 and 6) resulted in greater response to selection than training on the pure lines separately (Scenario 1) or on the pure lines combined (Scenario 2), although selection was based on GEBVC in all cases and no G × E interaction was included. Response to selection was greater when training was on crossbred animals for which both phenotypes and genotypes were available (Scenarios 5 and 6) than when training was on crossbreds for which only phenotypes were available and genotype probabilities based on their parents’ genotypes were used (Scenarios 3 and 4). In addition, when alternate heterozygotes Aa and aA could be distinguished in crossbred animals (Scenario 6), response to selection was greater than when they could not be distinguished (Scenario 5). Similarly, response to selection was greater when training was on genotype probabilities of crossbred animals for which alternate heterozygotes Aa and aA could be distinguished (Scenario 4) than when they were pooled together (Scenario 3). The phenotypic mean of crossbreds increased when breeds A and B had separate training populations (Scenario 1) compared to when a common training population consisting of animals from both breeds A and B was used (Scenario 2).

Finally, the difference in the amount of response to selection in the first generation compared to that in the subsequent generations is due to marker effects being estimated in the base generation only and to using these estimates to calculate the GEBVC in all subsequent generations. Thus, there was no retraining in each generation and GEBVC accuracy decreased over generations of selection, which caused a decline in genetic gain.

### Response to selection in purebreds

CP can be written as CP = BA + H, where BA denotes the breed average of pure lines and H the heterosis present in crossbreds [20]. Thus, the observed superiority of some scenarios may be due to a greater response in BA or in H, or in both. The cumulative response to selection averaged over breeds A and B for four generations of selection is in Table 3. Contrary to what was observed for response to selection for CP, response to selection within pure lines was greater when training was on pure lines although selection was based on GEBVC in all scenarios. Response to selection was greatest for Scenario 1 and smallest for Scenarios 3 and 4 with training on crossbred animals and using their genotype probabilities. However, when training was on phenotypes and genotypes of crossbreds (Scenarios 5 and 6), response to selection within pure lines was almost comparable to that for scenarios with training on pure lines. Similar to response for CP, response to selection within pure lines was greater when the alternate heterozygotes Aa and aA could be distinguished, i.e. Scenario 4 performed better than Scenario 3 and Scenario 6 performed better than Scenario 5.

**Table 3:** Mean phenotypic average of pure lines

	G1	G2	G3	G4	G5
Scenario 1	0.00	0.55	0.72	0.85	0.93
Scenario 2	0.00	0.50	0.67	0.78	0.86
Scenario 3	0.00	0.35	0.48	0.57	0.62
Scenario 4	0.00	0.42	0.54	0.63	0.70
Scenario 5	0.00	0.49	0.64	0.75	0.83
Scenario 6	0.00	0.49	0.66	0.77	0.87

Scenario 1: separate training in both breeds A and B; Scenario 2: training on a combined set of animals from both breeds A and B; Scenario 3: training on crossbred animals with phenotypes and genotype probabilities and it was assumed that the alternate heterozygotes Aa and aA were identical in crossbred animals; Scenario 4: training on crossbred animals with phenotypes and genotype probabilities and it was assumed that the alternate heterozygotes could be distinguished in crossbred animals. Scenario 5: training on crossbred animals with phenotypes and genotypes and it was assumed that the alternate heterozygotes were identical in crossbred animals. Scenario 6: training on crossbred animals with phenotypes and genotypes and it was assumed that the alternate heterozygotes could be distinguished in crossbred animals; standard errors of phenotypic means for simulated scenarios in generation 5 ranged from 0.03 to 0.04

### Heterosis in crossbreds

**Fig. 3:**
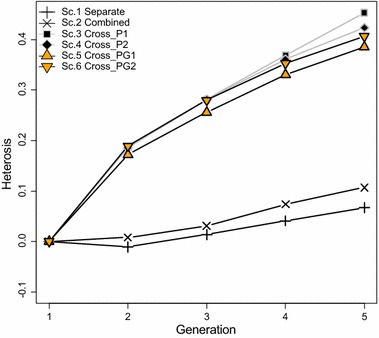
Heterosis in crossbreds. *Scenario 1*: separate training in both breeds A and B; *Scenario 2*: training on a combined set of animals from both breeds A and B; *Scenario 3*: training on crossbred animals with phenotypes and genotype probabilities and it was assumed that the alternate heterozygotes Aa and aA were identical in crossbred animals; *Scenario 4*: training on crossbred animals with phenotypes and genotype probabilities and it was assumed that the alternate heterozygotes could be distinguished in crossbred animals. *Scenario 5*: training on crossbred animals with phenotypes and genotypes and it was assumed that the alternate heterozygotes were identical in crossbred animals. *Scenario 6*: training on crossbred animals with phenotypes and genotypes and it was assumed that the alternate heterozygotes could be distinguished in crossbred animals

Heterosis refers to the superior performance of crossbred animals compared to the average performance of its purebred parents. Figure 3 shows the amount of heterosis over generations for the simulated scenarios. Total heterosis was calculated as the sum of heterosis at each locus based on $H = \mathop \sum \nolimits d_{l} (p_{A,l} - p_{B,l} )^{2}$, where $d_{l}$ is the dominance effect at QTL $l$, $p_{A,l}$ is the allele frequency at QTL $l$ in breed A, and $p_{B,l}$ is the allele frequency at QTL $l$ in breed B [20]. In all scenarios, the amount of heterosis increased over generations, however, the rate of increase differed among scenarios. The amount of heterosis in Scenarios 1 and 2 increased a little from generation 1 to 5, whereas it increased much more sharply in the other scenarios in which training was on crossbreds. Since heterosis depends on the differences in allele frequencies between two breeds, this increase suggests that training on crossbreds together with selection for CP result in diverging allele frequencies between the two breeds. This could be caused by allele frequencies moving in different directions in both breeds or by selection acting on different loci in the two breeds.

### Correlation of LD phase between breeds A and B

We estimated the correlation of LD phase between breeds A and B for the scenarios in which time of breed divergence (1, 50, 100, 200, 300 and 400 generations back) varied. In these scenarios, the correlation of LD phase for SNPs with a pairwise distance of 1 cM decreased as the number of generations since separation increased, i.e. correlations of 0.39, 0.22, 0.11, 0.05, 0.0 and −0.04 were obtained for scenarios including 1, 50, 100, 200, 300 and 400 generations since divergence, respectively.

### Effect of being able to distinguish between alternate heterozygotes

Table 4 shows the effect of being able to distinguish between alternate heterozygotes Aa and aA by comparing Scenarios 5 and 6, for different times since breeds A and B diverged. Time since divergence affected the relative ranking of Scenarios 5 and 6: when the two breeds were closely related (i.e. 1, 50 and 100 generations of separation), response to selection for CP was greater for Scenario 5 than for Scenario 6, when time since divergence increased to 200 generations, response to selection was almost the same for both scenarios and when time since divergence increased to 300 and 400 generations, response to selection was greater for Scenario 6 than for Scenario 5. Thus, these results showed that being able to distinguish between alternate heterozygotes Aa and aA (Scenario 6) increases response to selection when breeds have diverged a long time ago.

**Table 4:** Mean phenotype of crossbreds in generation five without or with distinguishing between both heterozygotes (Scenario 6 vs Scenario 5), for different times since divergence of the pure lines

Scenarios	Time since divergence				
	1	50	100	200	400
Scenario 5	1.21	1.32	1.33	1.20	0.94
Scenario 6	1.15	1.28	1.30	1.19	0.99

Scenario 5: training on crossbred animals with phenotypes and genotypes and it was assumed that the alternate heterozygotes were identical in crossbred animals. Scenario 6: training on crossbred animals with phenotypes and genotypes and it was assumed that the alternate heterozygotes could be distinguished in crossbred animals; standard errors of phenotypic means ranged from 0.02 to 0.03; note that the mean phenotype of crossbreds cannot be compared for different times since divergence, as they are results of distinct simulations

### Effect of the training population size on the response to selection

**Fig. 4:**
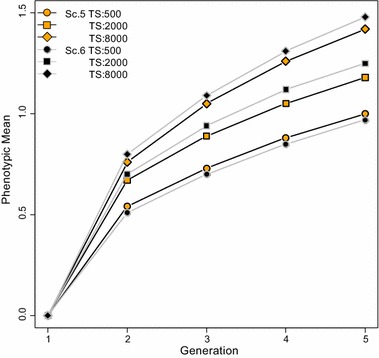
Cumulative response to selection for varying sizes of training populations. Training on crossbred animals with phenotypes and genotypes. *Scenario 5*: alternate heterozygotes Aa and aA were assumed identical in crossbred animals; *Scenario 6*: alternate heterozygotes could be distinguished in crossbred animals; TS stands for training population size of 500, 2000 and 8000; standard errors of phenotypic means ranged from 0.02 to 0.03

Figure 4 shows the cumulative response to selection in Scenarios 5 and 6 for varying sizes of the training population. To evaluate the impact of training population size on the relative ranking of Scenarios 5 and 6, 200 generations of divergence between breeds A and B were considered, since response to selection for these two scenarios was almost the same for this time since divergence and a training population size of 2000. As expected, response to selection with both scenarios increased as the size of the training population increased. However, the relative ranking of Scenarios 5 and 6 changed as the size of training population increased. If the size of the training population was 500, response to selection was greater for Scenario 5 than for Scenario 6, but with a 4- and 16-fold increase, response to selection was greater for Scenario 6 than for Scenario 5. Thus, these results showed that being able to distinguish between alternate heterozygotes Aa and aA was beneficial when the training population was large.

## Discussion

We investigated response to selection in crossbred performance in a two-way crossbreeding system of two related breeds for five generations. To estimate SNP effects, training was either on pure lines or crossbred animals, animals were selected on GEBVC, and there was no G × E interaction. Thus, the deviation of the purebred-crossbred genetic correlation $(r_{pc} )$ from 1 originated purely from dominance effects and differences in allele frequencies between the two purebred lines. We also investigated the effect of being able to distinguish between alternate heterozygotes Aa and aA in crossbred animals.

### Training on crossbred animals vs pure lines

A general finding of our study was that training on crossbred animals led to greater phenotypic response in crossbred animals compared to training on purebred lines. To identify the potential reasons for the superiority of training on crossbred animals, we partitioned the estimated breeding values (EBV) of animals in the pure lines into components due to additive and dominance effects (see Esfandyari et al. [15] for partitioning of breeding values). We found that, by training on crossbred animals, we could predict dominance effects and consequently breeding values of the animals in pure lines more accurately. Accuracy of EBV due to dominance effects when training was on crossbred animals was on average equal to 0.24, whereas when training was on pure lines, it was equal to 0.16 (see Additional file 1). This indicates that a higher prediction accuracy of dominance effects by training on crossbred animals is associated with a higher level of heterozygosity in the crossbred animals. Observed heterozygosity in the crossbred training population was 0.49 on average, which was higher than that found for the pure lines, i.e. 0.33 and 0.34 on average for breeds A and B, respectively. Logically, dominance effects can be predicted more accurately when the level of heterozygosity is higher.

In this study, we did not simulate environmental differences for purebred and crossbred animals. However, in practice, environments in which purebreds and crossbreds are kept are often different. Thus, selection of purebreds to improve crossbred performance in a commercial environment involves not only the $r_{pc}$ caused by non-additive genetic effects, but also a possible G × E interaction [7]. For instance, for a $r_{pc}$ of 0.8 due to dominance effects, it might be possible to reach the maximum accuracy (i.e. 1) by using an infinite amount of information on purebred animals under a dominance model. However, for a $r_{pc}$ of 0.8 only due to G × E interactions, the maximum achievable accuracy by using purebred information is 0.8. Thus, the mechanism that results in $r_{pc}$ less than 1 has an impact on response to selection under a dominance model. Nonetheless, by using crossbred data, it might be possible to reach maximum accuracy as well. Thus, a loss in genetic gain should be expected in the presence of G × E interactions compared to no G × E interactions. In other words, if a $r_{pc}$ less than 1 was partly due to G × E interactions, training on crossbred animals would be even more beneficial than the results show in this study.

Although training on crossbred animals led to greater response to selection in crossbreds, it requires the collection of data at the crossbred level. Since commercial crossbred animals are usually not individually identified and individual performances are not recorded, it might be difficult and expensive to collect phenotype and genotype data on crossbred individuals, whereas most breeding programs have routine phenotyping and genotyping of nucleus animals in the pure lines. If genotyping but not phenotyping of crossbred animals is a limiting factor, one could do training on crossbred animals with phenotypes and use genotype probabilities based on the genotypes of their purebred parents (Scenarios 3 and 4 in our simulation). With this strategy, it is possible to gain some of the benefits of crossbred training without genotyping crossbred animals (see Fig. 2). However, this strategy does require pedigree identification of crossbreds.

### Distinguishing between heterozygotes in crossbred animals

Our results showed that being able to distinguish between alternate heterozygotes Aa and aA in crossbred animals and to predict two distinct genetic values for these genotypes will lead to greater response to selection in crossbreds when the two purebred lines are distantly related. The reasons for this superiority are both differences in SNP and QTL frequencies between the two lines as well as differences in the amount and extent of LD between SNPs and QTL between the lines. Any difference in QTL and SNP frequencies and in LD between the pure lines can result in the two alternate heterozygotes at a SNP having different probabilities for a heterozygous QTL in the crossbreds. These differences suggest that one should distinguish between the two alternate heterozygotes in the crossbred when a dominance model is used for crossbred training.

Due to the genetic differences among the pure lines, we expected that being able to distinguish between alternate heterozygotes when training on crossbreds would always perform better. However, we found that this superiority was associated with two other factors; time since divergence of the two lines and number of records used in the training. The results showed that being able to distinguish between alternate heterozygotes was favourable only for distantly related lines (Table 4). In fact, in distantly related lines, the chance that recombination breaks down the shared ancestral haplotypes (and even reverses the LD phase) across the populations is greater. Hence, reverse LD phase between SNPs and QTL between the two lines for distantly related breeds can cause the two alternate heterozygotes at a SNP to have different QTL alleles in the crossbreds. Apparently, by predicting two genetic effects for alternate heterozygote genotypes, this difference in LD phase was captured and resulted in greater response to selection when pure lines were distantly related.

In our simulations, the number of records used in the training population also contributed to the observed differences in response for Scenarios 5 and 6. We found that with a small number of records used in the training data, response to selection was greater in Scenario 5 than in Scenario 6 (Fig. 4). This is probably due to the difference in number of effects that need to be predicted in the two scenarios. For Scenario 6, where alternate heterozygotes could be distinguished, four genotypic effects had to be predicted, whereas for Scenario 5 only three genotypic effects had to be predicted. Hence, because the number of effects to be predicted in Scenario 6 was greater, it was at a disadvantage over Scenario 5 with a small number of records. However, this disadvantage disappears as the training population size increases. In other words, as the number of records used for training increases, more information becomes available to predict the effects of SNPs and, given the sufficient number of records for training, differences in SNP effects between lines render Scenario 6 more advantageous. This result agrees with those of Ibanez-Escriche et al. [8], who showed that breed-specific allele substitution effects (BSAM) will have an advantage over across-breed allele substition effects, provided sufficient information is available for estimating the additional breed-specific effects.

Finally, it should be mentioned that a prerequisite for distinguishing between alternate heterozygotes in our study and for the implementation of BSAM in Ibanez-Escriche et al. [8] is that the purebred origin of SNP alleles in crossbreds is known, which may not be easily obtained for real data. Nevertheless, given the very high SNP density, it may be possible to trace alleles to ancestors accurately [23]. In a recent study, Bastiaansen et al. [24] suggested a method to determine breed origin of alleles in crossbreds using long-range phasing without the need for tracking pedigree relationships of crossbreds. Based on this method, it is not even necessary to have close relationships between the crossbred and genotyped purebred animals since long-range phasing will work even with distant purebred relatives of the crossbreds [24]. Hence, tools are available to distinguish between alternate heterozygotes, and also to take advantage of the associated benefits in practical situations.

### Simulation model

For reasons of computation time, simulation studies usually use a genome size which is smaller than that of most livestock species [25]. In our simulations, we used a genome with one chromosome 100 cM long. By assuming a phenotypic variance of 1, QTL on this chromosome result in an additive variance of 0.3. However, in real livestock genomes (e.g., a genome of 30 M for cattle), QTL on a chromosome of this length would cause an additive variance of only ~0.01. One consequence is that the sizes of the QTL effects in our simulation are substantially larger than those of real QTL, which means that the effects of simulated QTL were predicted more accurately than what may be possible with a real dataset. Daetwyler et al. [26] and Goddard [27] predicted that the accuracy of genomic selection depends on the parameter $\rho^{2} = \frac{{Th^{2} }}{ML}$, where $h^{2}$ is the heritability of the trait, $T$ is the number of records in the training data, $M$ is the effective number of loci per Morgan ($2Ne$), and $L$ is the genome size in Morgan. This relationship predicts that accuracy will be the same for all cases where $\rho^{2}$ is the same. So, under optimal conditions, a genome of 30 chromosomes of 1 M each requires 30 times as many training records to achieve the same accuracy as a genome with 1 chromosome 1 M long.

We did not check whether the number and effect of QTL or the density of SNPs affected the relative ranking of Scenarios 5 and 6. However, most probably by increasing the genome size and keeping all other parameters constant (i.e. SNP density, training population size and values of variance components), Scenario 6 would be at a disadvantage over Scenario 5 due to the greater number of effects that need to be predicted. This suggests that the benefit of being able to distinguish between alternate heterozygotes is expected to decrease as the genetic architecture becomes more polygenic. In addition, SNP density may affect the difference between Scenarios 5 and 6 as well. As SNP density increases, the model will include SNPs that are closer to the QTL. In a finite population, SNP alleles that are closer to the QTL will more accurately reflect the state of the QTL alleles [8]. Thus, as the SNP density increases, the need for distinguishing between alternate heterozygotes may be reduced.

Besides the small genome size that may cause overestimation of the accuracy of GEBV in our simulation, additive effects were sampled from a gamma distribution, which results in some QTL with a large effect that may account for a substantial part of the additive variance. Hence, genomic breeding values may be predicted more accurately than for a purely polygenic trait. In addition, in real populations, QTL effects may be line-dependent due to epistatic interactions, which may be negligible if selection is performed within a population but not if effects are estimated simultaneously for several populations. In fact, presence of epistatic interactions among genes may cause the lack of consistency across breeds. In this case, the effect of a particular QTL depends on the allelic frequency of genes it interacts with [28]. Since these frequencies can differ among breeds it results in breed-specific effects. Thus, combining animals from two breeds into a single training population may not be advantageous in the presence of substantial epistasis.

In this study, generation interval was the same for all scenarios with purebred or crossbred training. In other words, randomly selected sires of breed A in generation 2307 were mated to the dams of this breed to produce purebred offspring and also to the dams of breed B to produce crossbred offspring. Training was on randomly selected individuals from these offspring. Thus, scenarios with crossbred training did not require an additional generation compared to purebred training to create the training population.

In our simulations, regardless of whether training was on pure lines or crossbreds, a dominance model based on own performance of the animals was used to estimate the GEBVC for the selection candidates. However, an alternative approach would be to carry out training on pure lines based on the yield deviations of their crossbred progeny and to use an additive model to estimate breeding values. In other words, training can be done on purebred animals with genotypes and the mean phenotypes of their crossbred progeny can be used as response variable. We compared performance of Scenario 1 to such a scenario (referred to as additive scenario, hereafter) where training was on purebred animals, mean performance of crossbred progeny was used as response variable and an additive model was used to estimate GEBV. The size of the reference population for the additive scenario was 1000 within each pure line and each of the animals in the training set had 10 crossbred progeny. Result showed that using crossbred progeny information yielded a greater response to selection than using the animals’ own records although in both cases, training was on pure lines (see Additional file 2). Scenario 1 with a dominance model resulted in a smaller breed average response compared to the additive scenario which resulted in a smaller overall crossbred response. The superiority of the additive scenario is due to the increased accuracy of selection in pure lines by using crossbred progeny information. In fact, for Scenario 1, own performance of the animals in the training set was used as response variable, whereas for the additive scenario more information was available by using the mean performance of 10 crossbred progeny.

## Conclusions

Genomic selection can be very valuable in crossbreeding programs since it allows efficient selection for crossbred performance. To reach greater response to selection when crossing two distantly related lines, it is better to do training on crossbred animals rather than on pure lines to predict genetic effects. In addition, being able to distinguish between alternate heterozygotes in the crossbred training set by taking into account the breed origin of alleles increases response to selection, except when breeds are closely related and the reference population is small. Finally, our results showed that response to selection in crossbreds was greater when both phenotypes and genotypes were collected on crossbreds, compared to having only phenotypes on the crossbreds and genotypes on their parents.

## Additional files

10.1186/s12711-015-0155-z Partitioning accuracies of breeding values due to additive and dominance effects for Scenario 1 and Scenario 5.
10.1186/s12711-015-0155-z Comparison of Scenario 1 with an additive scenario. We compared the performance of Scenario 1 to an additive scenario (Scenario Additive) where training was on purebred animals, mean performance of crossbred progeny was used as response variable and an additive model was used to estimate genomic estimated breeding values. The size of the reference population for the additive scenario was 1000 within each pure line and each of the animals in the training set had 10 crossbred progeny.

## References

[1] Hartmann W (1992). Evaluation of the potentials of new scientific developments for commercial poultry breeding. World Poultry Sci J..

[2] Comstock RE, Robinson HF, Harvey PH (1949). A breeding procedure designed to make maximum use of both general and specific combining ability. Agron J..

[3] Bijma P, van Arendonk JAM (1998). Maximizing genetic gain for the sire line of a crossbreeding scheme utilizing both purebred and crossbred information. Anim Sci..

[4] Lo LL, Fernando RL, Grossman M (1993). Covariance between relatives in multibreed populations—additive-model. Theor Appl Genet.

[5] Wei M, van der Steen H (1991). Comparison of reciprocal recurrent selection with pure-line selection systems in animal breeding (a review). Anim Breed Abstr.

[6] Dekkers JCM (2007). Marker-assisted selection for commercial crossbred performance. J Anim Sci.

[7] Dekkers JCM, Chakraborty R (2004). Optimizing purebred selection for crossbred performance using QTL with different degrees of dominance. Genet Sel Evol..

[8] Ibanez-Escriche N, Fernando RL, Toosi A, Dekkers JCM (2009). Genomic selection of purebreds for crossbred performance. Genet Sel Evol..

[9] Zeng J, Toosi A, Fernando RL, Dekkers JCM, Garrick DJ (2013). Genomic selection of purebred animals for crossbred performance in the presence of dominant gene action. Genet Sel Evol..

[10] Kinghorn BP, Hickey JM, van der Werf JHJ. Reciprocal recurrent genomic selection for total genetic merit in crossbred individuals. In: Proceedings of the 9th World Congress on Genetics Applied to Livestock Production: 1–6 August 2010; Leipzig. Paper 36. 2010.

[11] Piyasatian N, Fernando RL, Dekkers JCM (2007). Genomic selection for marker-assisted improvement in line crosses. Theor Appl Genet.

[12] Daetwyler HD, Villanueva B, Bijma P, Woolliams JA (2007). Inbreeding in genome-wide selection. J Anim Breed Genet.

[13] Habier D, Fernando RL, Dekkers JCM (2007). The impact of genetic relationship information on genome-assisted breeding values. Genetics.

[14] Sonesson AK, Meuwissen THE (2009). Testing strategies for genomic selection in aquaculture breeding programs. Genet Sel Evol..

[15] Esfandyari H, Sorensen AC, Bijma P (2015). Maximizing crossbred performance through purebred genomic selection. Genet Sel Evol..

[16] Sargolzaei M, Schenkel FS (2009). QMSim: a large-scale genome simulator for livestock. Bioinformatics.

[17] Wellmann R, Bennewitz J (2012). Bayesian models with dominance effects for genomic evaluation of quantitative traits. Genet Res (Camb)..

[18] Wellmann R, Bennewitz J (2011). The contribution of dominance to the understanding of quantitative genetic variation. Genet Res (Camb)..

[19] Perez P, de los Campos G (2014). Genome-wide regression and prediction with the BGLR statistical package. Genetics..

[20] Falconer DS, Mackay TFC (1996). Introduction to quantitative genetics.

[21] Badke YM, Bates RO, Ernst CW, Schwab C, Steibel JP (2012). Estimation of linkage disequilibrium in four US pig breeds. BMC Genomics.

[22] Wei M, Vanderwerf JHJ (1994). Maximizing genetic response in crossbreds using both purebred and crossbred information. Anim Prod..

[23] Meuwissen THE, Goddard ME (2007). Multipoint identity-by-descent prediction using dense markers to map quantitative trait loci and estimate effective population size. Genetics.

[24] Bastiaansen JWM, Bovenhuis H, Lopes MS, Silva FF, Megens HJ, Calus MPL, editors. SNP Effects depend on genetic and environmental context. In: Proceedings of the 10th World Congress on Genetics Applied to Livestock Production: 17–22 August 2014; Vancouver. 2014. https://asas.org/docs/default-source/wcgalp-proceedings-oral/356_paper_10322_manuscript_1308_0.pdf?sfvrsn=2. Accessed 22 Sept 2014.

[25] Meuwissen THE, Hayes BJ, Goddard ME (2001). Prediction of total genetic value using genome-wide dense marker maps. Genetics.

[26] Daetwyler HD, Villanueva B, Woolliams JA (2008). Accuracy of predicting the genetic risk of disease using a genome-wide approach. PLoS One.

[27] Goddard M (2009). Genomic selection: prediction of accuracy and maximisation of long term response. Genetica.

[28] Carlborg O, Kerje S, Schutz K, Jacobsson L, Jensen P, Andersson L (2003). A global search reveals epistatic interaction between QTL for early growth in the chicken. Genome Res.

