# Supplementary material for: A crossbred reference population can improve the response to genomic selection for crossbred performance
Source: Genet Sel Evol. 2015 Sep 29;47:76. doi: 10.1186/s12711-015-0155-z (PMC4587753; doi:10.1186/s12711-015-0155-z)
Supplement: Supplementary file 1 — 10.1186/s12711-015-0155-z Partitioning accuracies of breeding values due to additive and dominance effects for Scenario 1 and Scenario 5. [file 12711_2015_155_MOESM1_ESM.pdf]

Additional file 1:

Partitioning accuracies of breeding values due to additive and dominance effects for Scenario 1 and Scenario 5.

Partitioning accuracies of breeding values due to additive and dominance effects

		Scenario 1			Scenario 5		
**Breed A**	**G**	**BV**	**Add**	**Dom**	**BV**	**Add**	**Dom**
	**1**	0.65	0.69	0.19	0.80	0.57	0.36
	**2**	0.49	0.56	0.18	0.61	0.40	0.22
	**3**	0.38	0.50	0.20	0.50	0.37	0.19
	**4**	0.30	0.47	0.19	0.43	0.34	0.19
	**5**	0.26	0.44	0.19	0.39	0.32	0.24
	**Mean**	0.42	0.53	0.19	0.54	0.40	0.24

		Scenario 1			Scenario 5		
**Breed B**	**G**	**BV**	**Add**	**Dom**	**BV**	**Add**	**Dom**
	**1**	0.64	0.71	0.15	0.79	0.61	0.36
	**2**	0.45	0.58	0.13	0.60	0.44	0.22
	**3**	0.35	0.50	0.11	0.49	0.38	0.19
	**4**	0.28	0.45	0.12	0.40	0.34	0.22
	**5**	0.26	0.43	0.15	0.32	0.30	0.22
	**Mean**	0.40	0.53	0.13	0.52	0.41	0.24

G: generation

BV: Accuracy of breeding values that is correlation between TBVC and GEBVC

Add: Accuracy of breeding values due to additive effects. **Dom:** Accuracy of breeding values due to dominance effects.

Scenario 1: Separate training in both breed A and B.

Scenario 5: Training on crossbred animals with phenotypes and genotypes. Two types of heterozygotes were assumed the same in crossbred animals.
