# Supplementary material for: A crossbred reference population can improve the response to genomic selection for crossbred performance
Source: Genet Sel Evol. 2015 Sep 29;47:76. doi: 10.1186/s12711-015-0155-z (PMC4587753; doi:10.1186/s12711-015-0155-z)

**Additional file 2:**

**Comparison of Scenario 1 with an additive scenario.**

We compared performance of Scenario 1 to a scenario (Scenario Additive) where training was on purebred animals, mean performance of crossbred progeny was used as response variable and an additive model was used to estimate genomic estimated breeding values. The size of the reference population for additive scenario was 1000 within each pure line and each of the animals in the training set had 10 crossbred progeny.

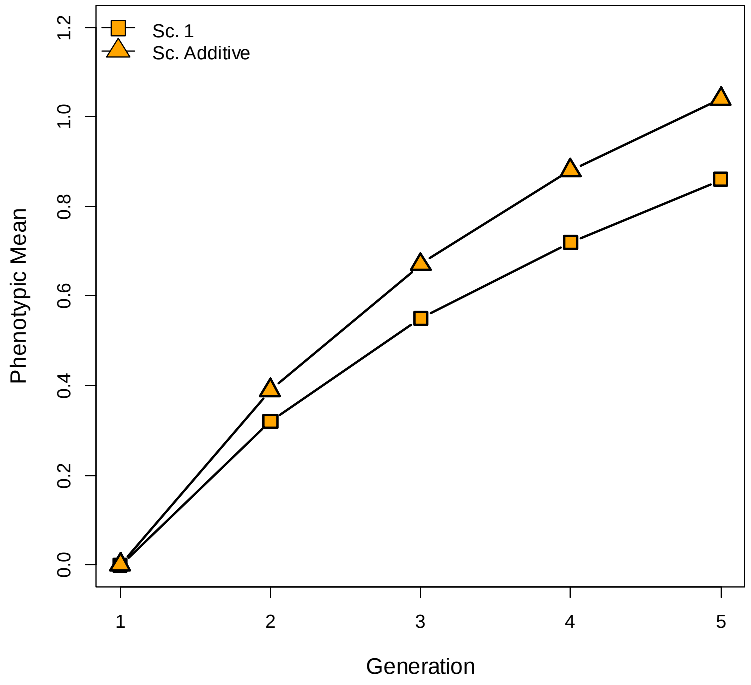

Supplement: Supplementary file 2 — 10.1186/s12711-015-0155-z Comparison of Scenario 1 with an additive scenario. We compared the performance of Scenario 1 to an additive scenario (Scenario Additive) where training was on purebred animals, mean performance of crossbred progeny was used as response variable and an additive model was used to estimate genomic estimated breeding values. The size of the reference population for the additive scenario was 1000 within each pure line and each of the animals in the training set had 10 crossbred progeny. [file 12711_2015_155_MOESM2_ESM.pdf]
